# TRANSITIONS INTO DEMENTIA: REFLECTIONS ON WALKING INTERVIEWS AND THE PHOTO-ELICITATION METHOD IN DEMENTIA STUDIES

**DOI:** 10.1093/geroni/igae098.0540

**Published:** 2024-12-31

**Authors:** Simone Niedoba, Frank Oswald

**Affiliations:** Goethe University Frankfurt, Frankfurt am Main, Hessen, Germany; Goethe University Frankfurt, Frankfurt am Main, Hessen, Germany

## Abstract

Environmental gerontology highlights behavioral and experiential processes between a person and the environment in relation to different outcomes. However, how people living with early dementia experience and shape their environment is still poorly understood and how these processes could be best assessed is still being debated. Walking interviews promise to combine observation of everyday interactions, and discussion of meanings of environments. Pictures taken by participants along the way may give further insights into personal relevant environments. During nine walking interviews people living with early dementia were accompanied on a routine walk through their local environment. Participants decided where to go and were invited to take pictures of their environment, which were discussed at the end of the interview. The interviews highlight the importance of environments for the experience of living with dementia. During the interviews real-time interactions with places, objects, and people were observed and functioned as prompts for discussion, highlighting not only how people living with dementia shape their environment, thus conducting subtle forms of participation, but also how environments were connected to memories and identity. The combination of walking interviews and the photo-elicitation method allows to assess relevant person-environment exchange processes, such as agency and belonging, in early dementia. Moving through familiar environments can enhance a comfortable and trustful atmosphere in which personal reflections on living with dementia are shared. In the talk we will critically reflect on the experiences with these methods, including challenges, limitations and ethical considerations, and discuss how these methods could benefit future research.

